# A systematic review on the association between the *Helicobacter pylori vacA i* genotype and gastric disease

**DOI:** 10.1002/2211-5463.12046

**Published:** 2016-04-05

**Authors:** Xian Liu, Bangshun He, William C. Cho, Yuqin Pan, Jie Chen, Houqun Ying, Feng Wang, Kang Lin, Hongxin Peng, Shukui Wang

**Affiliations:** ^1^ Central LaboratoryNanjing First HospitalNanjing Medical UniversityJiangsuChina; ^2^ Department of Clinical OncologyQueen Elizabeth HospitalHong KongChina; ^3^ Department of Life SciencesNanjing Normal UniversityJiangsuChina; ^4^ Medical CollegeSoutheast UniversityNanjingJiangsuChina

**Keywords:** Gastric cancer, *Helicobacter pylori*, meta‐analysis, *vacA i* genotype

## Abstract

*Helicobacter pylori* (*H. pylori*) has been recognized as a cause of gastrointestinal diseases and progress of the pathology of gastrointestinal diseases is related to the genotype of *H. pylori*. Published studies have indicated that the *H. pylori vacuolating cytotoxin gene A* (*vacA*) *i1/i2* genotype is associated with peptic ulcer disease (PUD) and gastric cancer (GC), but their conclusions are inconsistent. This study aimed to further assess the risk of *vacA i* gene for PUD and/or GC. A systematic search was conducted across three main electronic databases (PubMed, Web of Science, and CNKI). A meta‐analysis was then performed on the pooled data of the published articles to estimate the overall influence of *vacA i* polymorphisms on PUD and/or GC by crude odds ratio (OR) with 95% confidence intervals (CI). The reliability of the results were confirmed by publication bias and sensitivity analysis of included studies. A total of 14 studies were selected according to the specific inclusion and exclusion criteria. The pooled results revealed that patients with GC were more vulnerable to infection by *H. pylori i1* genotype (OR = 5.12; 95% CI: 2.66–9.85; *P* < 0.001) than those with chronic gastritis or nonulcer disease. Moreover, the results of subgroup analysis indicated that the *i1* genotype of *H. pylori* was associated with an increased GC risk (OR = 10.89; 95% CI: 4.11–20.88; *P* < 0.001) in the Middle Asian population. The *H. pylori vacA i1* genotype is associated with an increased GC risk, especially in the Middle Asian population.

**Abbreviations**
*babA2*: blood group antigen‐binding adhesion protein A2
*cagA*: cytotoxin‐associated gene A
CG: chronic gastritis
CI: confidence interval
DU: duodenal ulcer
*dupA*: duodenal ulcer promoting
E: East Asia
GC: gastric cancer
GU: gastric ulcer
*H. pylori*: *Helicobacter pylori*
χ^2^: Chi square value
M: Middle Asia
NUD: nonulcer disease
*oipA*: outer inflammatory protein gene
OR: odds ratio
PUD: peptic ulcer disease
U: Europe
vacA: the vacuolating cytotoxin gene A

It is estimated that half of the global population have *Helicobacter pylori* (*H. pylori*) and this bacteria is proven to be related to gastrointestinal diseases, including chronic gastritis, peptic ulcers, and gastric cancer (GC) 1. Therefore, *H. pylori* has been categorized as group I carcinogen by the International Agency for Research on Cancer in 1994 2. GC is the fifth most common cancer and the third leading cause of cancer‐related deaths in the world 3. Although the global incidence rate of GC is decreasing, it is still a heavy burden to public health in some developing countries 3.

A previous study suggested that the increased risk of GC was determined by the interaction of *H. pylori* in the host and was affected by the genetic variation in the patient 4. It is generally accepted that the pathogenicity of *H. pylori* is determined by genetic variations associated with severe gastroduodenal diseases [including GC and peptic ulcer disease (PUD)], such as the presence of cytotoxin‐associated gene A (*cagA*), vacuolating cytotoxin gene A (*vacA i*,* s*,* m*,* d*), duodenal ulcer promoting (*dupA*), blood group antigen‐binding adhesion protein A2 (*babA2*), and outer inflammatory protein gene (*oipA*). Moreover, *vacA* gene is deduced to have a biological influence on the host gastric epithelial cell, including apoptosis induction, increased permeability of the epithelial monolayer, hexametric pores forming, and suppressed immunity 4, 5. *H. pylori vacA* gene has two genotypes within its signal (*s1*,* s2*), middle *(m1*,* m2*), deletion (*d1*,* d2*), and intermediate regions (*i1*,* i2*). Accumulated evidence suggest that *H. pylori* strains with *s1* lead to stronger cytokine activity than those with *s2*, indicating the *m1* strains to be more virulent than *m2* 6, 7. In addition, the *d1* genotype is found to be involved in the progression of carcinogenesis 8. Among these genotypes, *vacA i* has been widely investigated since it was first identified by Rhead *et al*. in 2007 9. In Asia, some studies have reported that the *H. pylori vacA i1* genotype may increase the risk of GC in the Iran population 10, 11. In Europe, it has been reported that *vacA i1* is associated with GC 10 and PUD 12, respectively. However, *vacA i1* is not the only factor to predict the pathological progression of GC 13 and the susceptibility of *vacA i1* to severe gastric disease remains unclear. This study aims to evaluate the association of the *H. pylori vacA i1* and *i2* genotypes with the risk of PUD and GC.

## Materials and methods

### Study sources and article search

To identify relevant articles, a comprehensive systematic search across three databases (PubMed, Web of Science, and a Chinese digital database CNKI) was performed to identify studies published until June 15, 2015, using the words ‘*Helicobacter pylori*’, ‘*H. pylori*’, ‘genotypes’, ‘*vacA i1*’, ‘intermediate region’, and ‘*vacA* gene’ with Boolean operators (NOT, AND, OR). Furthermore, a manual search was also performed to obtain substantial relevant studies by reviewing all references in the eligible articles. All analysis methods were in accordance with the preferred reporting items for systematic review and meta‐analyses (PRISMA) statement 14.

### Study selection

All included studies must meet the following inclusion criteria: (a) study reported the infection of *vacA i1* and/or *i2*; (b) case–control studies concerned about PUD [including gastric ulcer (GU) and duodenal ulcer (DU)] and/or GC with *H. pylori*‐positive control group [nonulcer disease (NUD) or chronic gastritis (CG)]; (c) with sufficient original data or information to assess the odds ratio (OR) and with 95% confidence interval (CI); (d) published in English or Chinese. On the other hand, the exclusion criteria include: (a) review articles; (b) case studies; (c) animal or functional experiments; (d) duplicate publications; (e) lack of raw data even after contacting the author; (f) conference proceedings; (g) literatures not written in English or Chinese.

### Data extraction

Two independent authors (Xian Liu and Bangshun He) extracted raw data from the eligible studies. For any discrepancy, a final consensus was sought after discussion with the research team. Data concerning the first author's last name, publishing year, the ethnicity or continent of the cases and controls, the amplification primer for detection, adult or children of subjects, and the disease of the control group were extracted from the studies.

### Statistical analysis

state software version 11.0 (STATA Corp., College Station, TX, USA) was applied for statistical analysis. The association between the presence of the *H. pylori vacA i1* genotype and the risk of PUD or GC was assessed by crude OR with 95% CI. A *P* value < 0.05 was considered as statistically significant. The Q test (Chi square value, χ^2^) was used to evaluate heterogeneity (*P* < 0.05 indicated a significant heterogeneity between studies). The Mantel–Haenszel method was used to calculate the pooled ORs of fixed‐effects model in case if there was no significant heterogeneity among studies (*P* > 0.05 or χ^2^ < 50%). On the other hand, the DerSimonian and Laird method was applied for random‐effects model (*P* < 0.05 or χ^2^ > 50%) 15. Sensitivity analysis was also conducted to explore the origin of significant heterogeneity. Beside, subgroup analysis was performed to explore the effects of geographical region, ulcer location, and control resources. In addition, publication bias was evaluated by funnel plots and the quantitatively evaluation was conducted by the Egger's test, significant publication bias was reckoned with a *P* value < 0.1 14.

## Results

### Included studies

**Figure 1:**
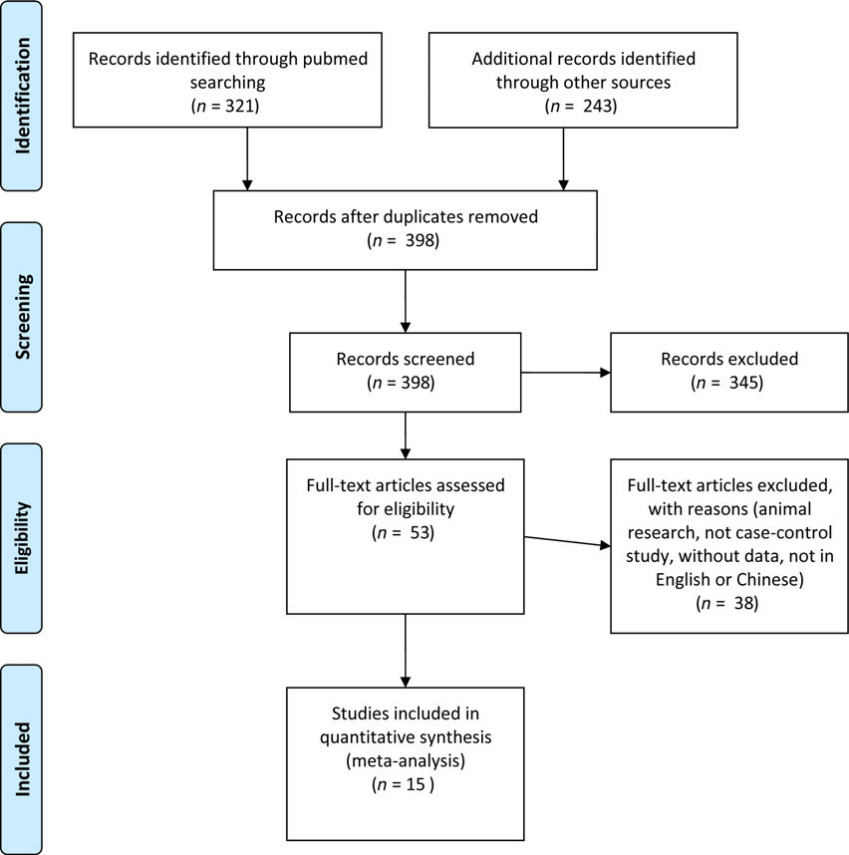
Flow diagram for the studies included in the meta‐analysis.

A total of 15 studies 9, 10, 11, 12, 13, 16, 17, 18, 19, 20, 21, 22, 23, 24, 25 were identified, including 13 full papers and two correspondence (a short paper type with available data) articles 22, 25, in which 14 were written in English and one in Chinese (Fig. 1). In the included studies, a total of 2667 patients were infected with the *H*. *pylori vacA i1* or *i2* genotype. Among these patients, a total of 85 subjects presented both the *vacA i1* and *i2* genotypes. These patients were counted in both of the *i1* and *i2* genotypes to reduce intergroup heterogeneity and to accurately detect *i2* pathogenicity. The main characteristics and specific values of each study were shown in Table 1.

**Table 1:** Characteristics of studies included in the meta‐analysis

Author/year	Country	Race	CG or NUD (*i1/i2*)	PUD (*i1/i2*)	GU (*i1/i2*)	DU (*i1/i2*)	GC (*i1/i2*)
Kim/2014 13	South Korea	E	75/10	–	103/15	102/10	121/15
Markovska/2011 20	Bulgarian	U	14/16	27/7	–	–	–
Mottaghi/2014 16	Iran	M	19/29	6/17	–	–	21/3
Memon/2014 11	Iraq	M	36/29	–	–	27/5	15/1
Jiang/2013 17	China	E	87/5	63/1	–	–	21/0
Ferreira/2012 18	Portugal	E	24/64	–	–	–	45/5
Yordanov/2012 19	Bulgarian	U	48/16	89/63	–	–	–
Panayotopoulou/2010 21	Greece	U	45/34	46/19	–	–	–
Douraghi/2010 22	Iran	M	75/90	–	–	–	42/6
Jang/2010 23	South Korea	E	99/4	–	41/1	46/3	30/0
Yakoob/2009 24	Pakistan	M	9/113	–	27/3	27/5	35/6
Douraghi/2009 10	Iran	M	61/78	20/14	103	–	30/4
Basso/2008 12	Italy	U	31/40	30/15	–	–	35/9
Ogiwara/2008 25	Japan	E	118/4	–	–	51/4	81/2
Rhead/2007 9	Iran	M	19/29	–	–	102	28/16

CG, chronic gastritis; DU, duodenal ulcer; E, East Asia; GC, gastric cancer; GU, gastric ulcer; M, Middle Asia; NUD, nonulcer disease; PUD, peptic ulcer disease; U, Europe.

All the included studies used either nonulcer disease or chronic gastritis as the control group. The study conducted by Yakoob *et al*. 24 had a significant heterogeneity comparing with the other included studies, which caused dramatic publication bias and affected the credibility of the outcome. Therefore, we excluded this study and the remaining 14 studies with 2445 patients were pooled for meta‐analysis. A standard PCR assay was performed by all of the included studies for the detection of the *vacA i* genotype. The study subjects were divided into East Asian, Middle Asian, and European populations according to geographical location. Considering the difference of geographic location, data from different regions in the same study were retrieved separately for subgroup analysis. The main characteristics and specific values of each subgroup were shown in Table 2.

**Table 2:** The main results of pooled analyses in exploring GC and PUD risk with the *H. pylori i1*/*i2* genotype

	Variables	Literatures, *n*	Patients, *n* (*i1/i2*)	Controls, *n* (*i1/i2*)	OR	95% CI	*P*_*heterogeneity*_	χ^*2*^ (%)
PUD	Total	11	651/174	804/289	1.38	0.87–2.17	0.002	61.1
	GU	2^a^	144/16	174/14	0.99	0.45–2.19	0.625	0
	DU	4	226/22	324/47	1.24	0.46–3.33	0.045	62.7
	PU	7	281/136	305/228	1.52	0.79–2.93	0.001	72.7
	Total	11	651/174	807/279	1.41	0.87–2.29	0.001	66.6
	E	4	406/34	553/37	1.00	0.60–1.66	0.421	0
	U	4	192/104	138/106	1.68	0.65–4.37	0.000	83.2
	M	3	53/36	116/136	1.64	0.57–4.76	0.027	72.3
GC	Total	11	504/67	653/495	**5.12**	**2.66–9.85**	0.001	67.4
	E	5	298/22	403/87	3.19	0.63–16.21	0.000	81.9
	M	5	171/36	219/368	**6.64**	**3.57–12.34**	0.201	33.1
	U	1	35/9	31/40	**5.02**	**2.10–11.97**	–	–
	NUD	6	317/44	384/240	**3.74**	**1.59–8.81**	0.004	71.3
	CG	5	152/17	260/142	**8.69**	**3.82–19.75**	0.165	38.5

CG, chronic gastritis as control source; CI, confidence interval; DU, duodenal ulcer; E, East Asia; GC, gastric cancer; GU, gastric ulcer; χ^2^, Chi‐square value; M, Middle Asia; NUD, nonulcer disease as control source; OR, odds ratio; PU, peptic ulcer; PUD, peptic ulcer disease; U, Europe. Statistically significant results were in bold.

a Two literatures, Kim and Jing, conducted both GU and DU cases resulting in a total of 11 studies.

### Association between the *vacA i1* genotype and PUD

**Figure 2:**
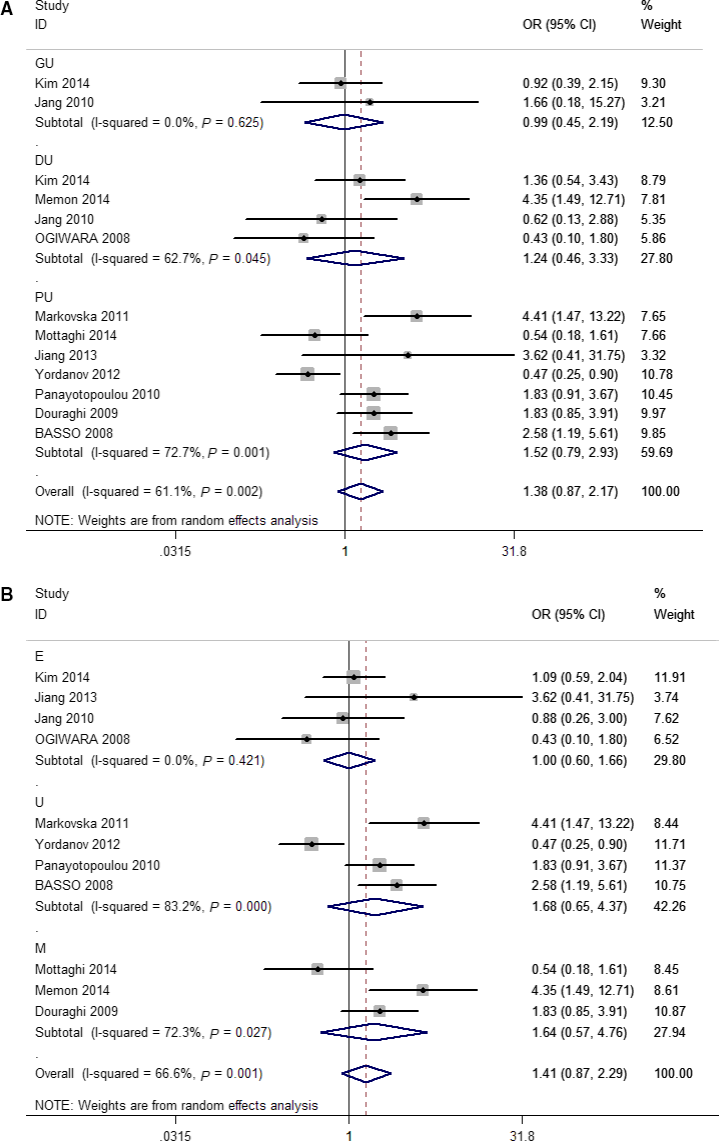
Forest plots of effect estimates for *vacA* polymorphism and PUD risk (*i1 vs i2*, overall and subgroup analysis). For each study, the estimation of odds ratio (OR) with 95% confidence interval (CI) is plotted with a box and a horizontal line. Filled diamond means pooled OR with 95% CI. DU, duodenal ulcer; E, East Asia; GU, gastric ulcer; M, Middle Asia; PU, peptic ulcer; U, Europe.

Meta‐analysis for the pooled results from 11 studies [10, 11, 12, 13, 16, 17, 19, 20, 21, 23, 25] showed no significant association between the *H. pylori vacA i1* genotype and PUD (pooled OR = 1.38, 95% CI: 0.87–2.17; *P*_heterogeneity_ = 0.002, χ^2^ = 61.1%). In the subgroup analysis for ulcer location (GU and DU), no significant association was observed between the *H. pylori i1* genotype and GU (pooled OR = 0.99, 95% CI: 0.45–2.19) or DU (pooled OR = 1.24, 95% CI: 0.46–3.33) (Fig. 2A). When grouping studies according to geographical region (East Asia, Middle Asia, and Europe), the pooled results showed that the *H. pylori i1* genotype was not associated with PUD in different region (East Asia: OR = 1.00, 95% CI: 0.60–1.66; Middle Asia: OR = 1.64, 95% CI: 0.57–4.76; Europe: OR = 1.68, 95% CI: 0.65–4.37) as shown in Fig. 2B.

### Association between the *vacA i1* genotype and GC

**Figure 3:**
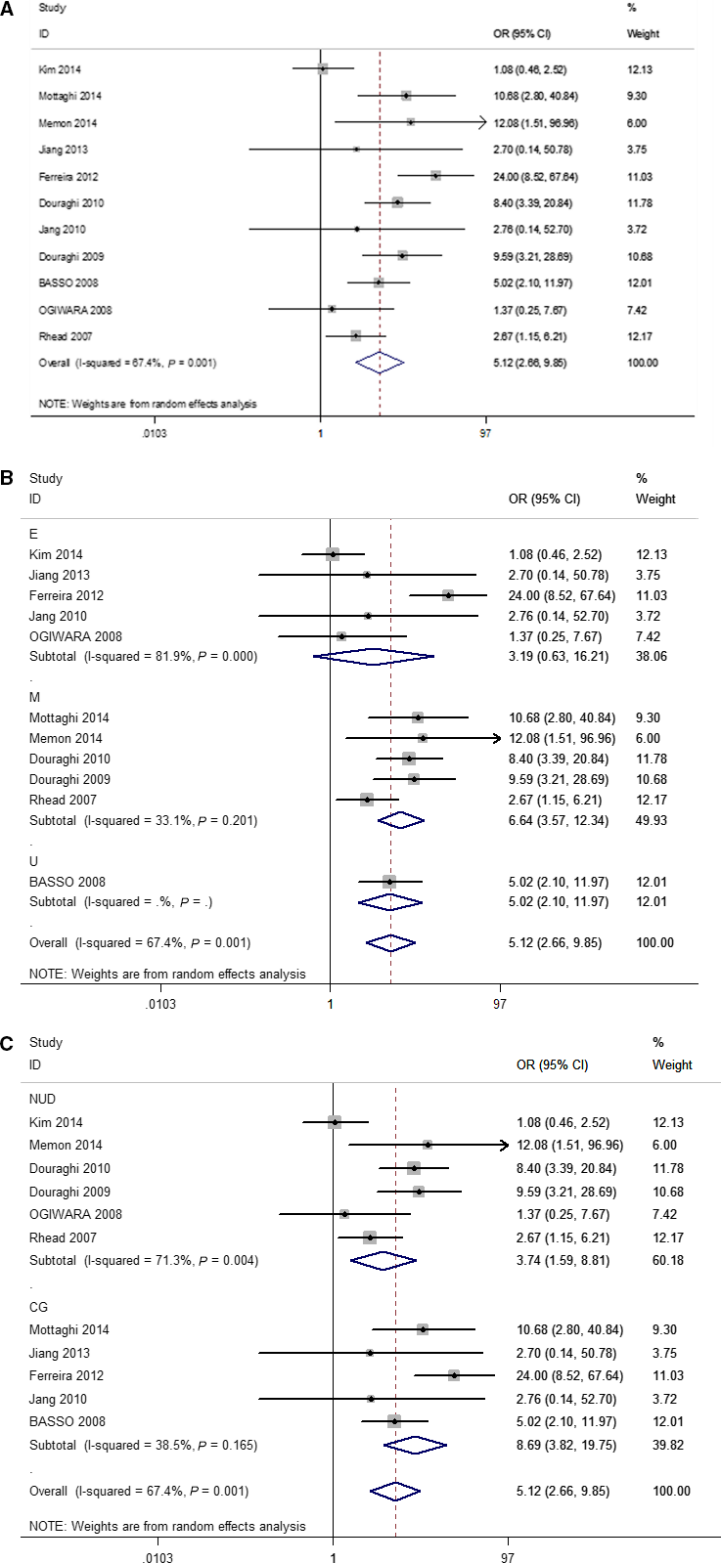
Forest plots of effect estimates for *vacA* polymorphism and GC risk (*i1 vs i2*, pooled analysis). For each study, the estimation of odds ratio (OR) with 95% confidence interval (CI) is plotted with a box and a horizontal line. Filled diamond means pooled OR with 95% CI. CG, chronic gastritis; E, East Asia; M, Middle Asia; NUD, nonulcer disease; U, Europe.

The association between the *H. pylori vacA i1* genotype and GC was assessed for 11 studies [9, 10, 11, 12, 13, 16, 18, 22, 23, 25] and the results indicated that the *H. pylori vacA i1* genotype was associated with an increased risk of GC (pooled OR = 5.12, 95% CI: 2.66–9.85*, P*_heterogeneity_ = 0.001, χ^2^ = 67.4%) as shown in Fig. 3A. Furthermore, ethnicity subgroup analysis revealed that the *H. pylori vacA i1* genotype was associated with an increased GC risk in the Middle Asian population (OR = 6.64, 95% CI: 3.57–12.34), but not in the East Asian population (OR = 3.19, 95% CI: 0.63–16.21) as shown in Fig. 3B. In addition, the subgroup analysis by the origin of control (NUD and CG) revealed that individuals with infection of *H. pylori i1* have an increased GC risk in both the NUD (OR_NUD_ = 3.74, 95% CI: 1.59–8.81) and CG (OR_CG_ = 8.69, 95%CI: 3.82–19.75) control groups as shown in Table 2 and Fig. 3C.

### Publication bias and sensitivity analysis

**Figure 4:**
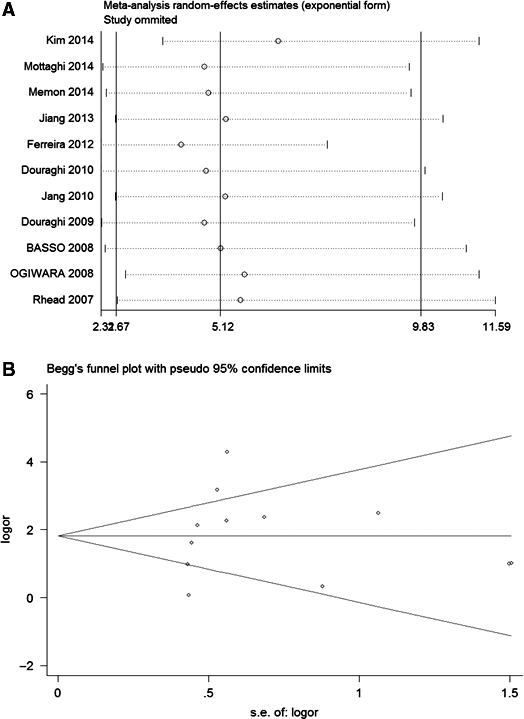
(A) Publication bias test for all included studies concerning GC risk (*i1 vs i2*). Each circle represents an independent study for the indicated association. Log[odds ratio (OR)]: natural logarithm of OR. Horizontal line shows the mean effect size. SE represents the standard error. (B) Sensitivity analysis of the effect of individual study on the pooled ORs for *vacA i1*/*i2* and GC risk. Each study is represented by one circle. The horizontal line represents the pooled effect estimate.

Funnel plots were used to evaluate the publication bias of the association between the present of the *vacA i1* genotype and GC qualitatively. The shape of the funnel plot did not reveal any evidence of obvious asymmetry (*t* = 0.048, *P* = 0.639). The Forest plot demonstrated that no significant publication bias was observed for the studies of GC. Sensitivity analysis revealed that no single study presented a dramatic influence for the final conclusion as shown in Fig. 4A,B.

## Discussion

This meta‐analysis has confirmed that the *vacA i1* genotype is closely related with an increased risk of GC in the Middle Asian population. Moreover, the pooled analysis also indicates that the *vacA i1* genotype has no association with the occurrence of PUD and GC except in Middle Asia. These findings suggest that GC is associated with the infection of *H. pylori* strains harboring the *vacA i1* genotype. The conclusions of four included studies 13, 17, 18, 23 based on Asian population are in agreement with this study. However, this trend does not apply to all of the Asian population 9. The difference in the association between *H. pylori vacA i1* and GC among different parts of Asia may be caused by an interaction between the host and other factors.

The results of this study are consistent with previous studies conducted in the Asian population 10, 11, but not in the European population 13, which may be explained by the fact that almost all strains in the East Asian populations are the *i1* genotype, whereas only approximately 50% of strains in the European and Middle East populations are the *i1* genotype 25. In addition, the dietary habit and host gene polymorphism can partly explain the differences 3.

Although *vacA* exists in all *H. pylori* strains, it can hardly induce severe cytoplasmic vacuolation 26. In fact, Rhead *et al*. 9 first proposed the *H. pylori vacA i1* genotype as an independent risk factor for gastric disease. The polymorphic differences in the *i*‐region may affect vacuolation activity, which present no association with other *vacA* genotype. Thereafter, many studies tried to find the association among the *vacA i1*,* m1*,* s1* genotype and diseases of the digestive tract. A similar meta‐analysis has concluded that *vacA s1* and *m1* exert an increased GC risk than their allele gene *s2* (OR 5.32, 95% CI: 2.76–10.26) and *m2* (OR 2.50, 95% CI: 1.67–3.750), respectively 27. Interestingly, the data of *s1 vs s2* is exactly similar to our result (OR 5.12, 95% CI: 2.66–9.85), which indicates that *vacA s1* and *i1* may share synergetic effects in the process of carcinogenesis. Argent *et al*. 28 also found that *vacA i1* might not be considered as an independent variable in gastric pathogenesis, they proposed that genetic variation within each virulence factors might affect the function of their own products.

The *vacA i1* and *i2* genotypes of *H. pylori* is the target of disease prevention and drug development. Winter *et al*. 29 found that the strains with *vacA i1* remained an increased colonization success rates and planting densities when comparing with the *i2* genotype. Moreover, the pathogenesis function of *vacA i* was further confirmed by Ogiwara *et al*. 30, who demonstrated that the *vacA i1* strains infection induced severer inflammatory cell infiltration in gastric epithelial cell when compared with the *i2* genotype. In addition, in the mouse‐colonizing *H. pylori* strain with *cagA* negative, *vacA* demonstrated an independent effect on the induction of inflammation and metaplasia, indicating that *s1/i1* type of *vacA* is the most pathogenic genotype. To a certain extent, *H. pylori vacA* may drive metaplasia through a different mechanism, which might explain why a different model has obtained a different conclusion. Based on this, the presence of specific *i*,* s*,* m*‐region polymorphisms is identified as a significant risk factor for digestive disease in certain populations.

Currently, it is well known that the infection rate of *H. pylori* is determined by living environment, dieting habits, and geographic regions. *H. pylori* is an important cause for PUD and GC. However, the pathogenicity was modified by the genotype of *H. pylori*. Therefore, it is urgently needed to identify the specific genotype of *H. pylori* isolated from the PUD and GC patients, which may significantly reduce the costs of screening and prevention for PUD and GC 31, 32. Indeed, the prediction role of the *vacA i* genotype may be varied in different pathogenic mechanisms. We could not conclude that the *vacA i* genotype statues are more closely associated with PUD and GC based on the limited samples in this study. Nevertheless, we anticipate that the *vacA i1* and *i2* genotypes may be a potential indicator for the risk of PUD and GC among patients infected with *H. pylori* strains.

There are some limitations in this meta‐analysis. Firstly, the languages of the included studies are only limited to English and Chinese, which may contribute to selection bias. Secondly, the control groups are constituted of CG, NUD, and functional dyspepsia. Some studies do not clarify whether the control group has excluded other disease or not, which may result in an underestimate of the effect of *H. pylori* strains. Thirdly, there is a lack of published reports with a focus on European and American populations, which prevents us from making a generalizable conclusion. Finally, other important data such as age, gender, family history, dieting habits, and other virulent factors are not available to investigate the association between *vacA i* gene and these factors.

## Conclusion

This is the first meta‐analysis evaluating the association between the *vacA* i genotype and the risk of GC and PUD. Our results demonstrate that *H. pylori vacA i1* gene is associated with an increased risk of GC comparing with the CG and PUD controls.

## Author contributions

XL extracted the data and wrote the manuscript. BH extracted the data and revised the manuscript. HY and YP searched and screened the eligible articles. FW and KL conducted the statistics. HP prepared Tables and Figures. SW designed the study. SW and WCC reviewed, revised, and approved the manuscript.
